# Spontaneous Cerebrospinal Fluid Leakage at the Clivus: Minimally Invasive Surgery Without External Autograft Harvesting

**DOI:** 10.7759/cureus.48009

**Published:** 2023-10-30

**Authors:** Kosuke Takabayashi, Junya Iwama, Katsumi Takizawa

**Affiliations:** 1 Otorhinolaryngology, Japanese Red Cross Asahikawa Hospital, Asahikawa, JPN; 2 Otorhinolaryngology, Sapporo Medical University School of Medicine, Sapporo, JPN; 3 Neurosurgery, Sassa General Hospital, Nishitokyo, JPN; 4 Neurosurgery, Japanese Red Cross Asahikawa Hospital, Asahikawa, JPN

**Keywords:** skull-base, nasoseptal flap, minimally invasive, endoscope, artificial material

## Abstract

Spontaneous cerebrospinal fluid (CSF) leakage at the clivus is rare. In previous reports, reconstructive materials used to treat such leakage were typically autografts. Considering the pathology, rigid reconstruction is preferred. We here describe a case of spontaneous CSF leakage at the clivus with multiple bony defects. In this case, in addition to using artificial material instead of autografts, such as fat or fascia, that require additional extranasal invasive harvesting site, a rigid material layer of septal cartilage and bone was also used, enabling more stable multilayer reconstruction. One month postoperatively, computed tomography revealed that the bony defect at the clivus had been well reconstructed. All nasal structures were preserved, and the nasoseptal flap was well engrafted. At eight months post-surgery, the patient remained in good condition. This method allows minimally invasive repair of the leaking clivus, according to the underlying pathophysiology.

## Introduction

Spontaneous cerebrospinal fluid (CSF) leakage is relatively rare, accounting for approximately 5-10% of all types of CSF leaks [1]. Such leaks at the clivus are particularly rare [1,2]. These are thought to be related to excessive aerification of the sphenoid sinus, chronic intracranial hypertension, and pulsation of the basilar artery [2-5]. Various transsphenoidal reconstruction methods have been reported previously [1-13].

Most fistulas related to CSF leakage at the clivus are only a few millimeters in diameter [2,3,13], and previous reports have used fat or fascia for their reconstruction, which requires the invasion of a site other than the nose for the harvesting of autograft material [1-13].

Here, we report a case of CSF leakage at the clivus, in which we utilized artificial materials, nasal septal cartilage, and bone for reconstruction, in a method that is less invasive than those previously reported. Additionally, two small bony defects were resected and reshaped to create one large defect. This approach allowed for precise delineation of the bone and dura layers, resulting in successful rigid reconstruction. This case highlights the utility of such minimally invasive reconstruction with precise layer identification.

## Case presentation

A 72-year-old woman with a body mass index of 18.6 kg/m^2^, who had been experiencing serous rhinorrhea for the past month presented to our Neurology Department with a headache and high fever. A lumbar puncture revealed bacterial meningitis, and the patient was subsequently referred to the Otorhinolaryngology Department for identification of the source. In the absence of a rhinologist, another otolaryngologist attended to the patient using an endoscope and evaluated her computed tomography (CT) images but could not determine the cause. The patient was discharged after the symptoms of bacterial meningitis improved with conservative treatment.

**Figure 1:**
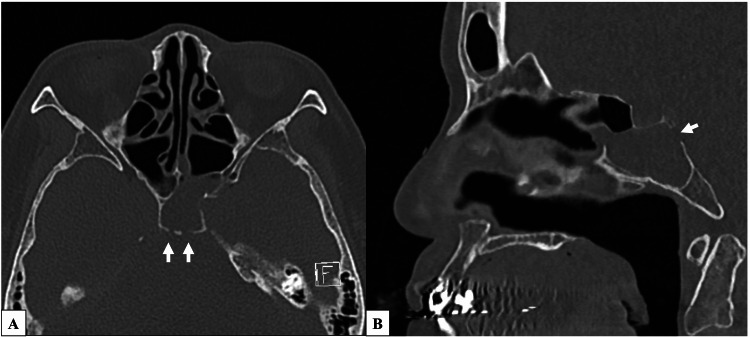
Preoperative computed tomography (CT) images of the area around the clivus. Computed tomography (CT) scan of a 0.5-mm slice revealed multiple bony defects in the upper clivus. White arrows indicate the bony defects.

**Figure 2:**
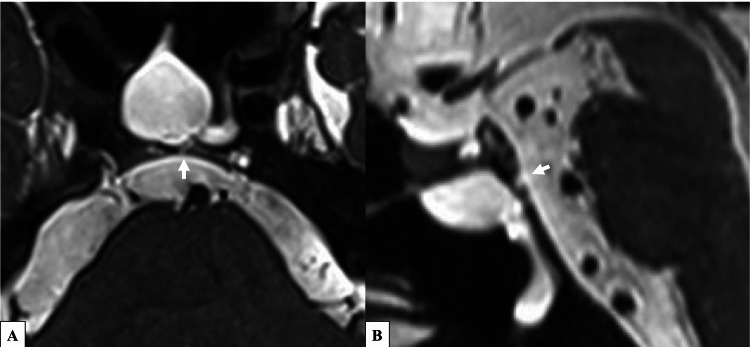
Preoperative magnetic resonance (MR) image of the area around the clivus. MR cisternography reveals a dural defect near the area of the bony defect identified by computed tomography. The white arrows indicate the dural defects.

Four months after discharge, she developed bacterial meningitis again. She was readmitted to the Neurology Department and subsequently referred to the Otorhinolaryngology Department. The patient also had a persistent serous nasal discharge. When this discharge was collected and analyzed, the glucose level in the fluid was 93 mg/dl, strongly suggesting CSF leakage. A review of thin-slice CT images revealed multiple bony defects in the clivus (Figure 1). Magnetic resonance cisternography revealed a suspected dural defect at one of the bony defect sites on the CT images (Figure 2). Based on these findings, a diagnosis of spontaneous CSF leakage at the clivus was made, and transnasal endoscopic skull base reconstruction by a cross-department team consisting of an otorhinolaryngologist and neurosurgeons was planned.

**Figure 3:**
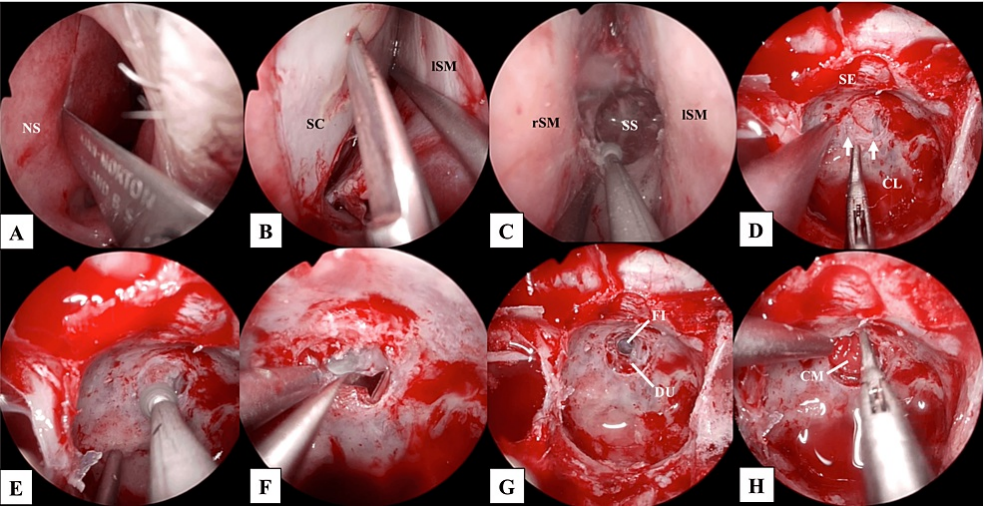
Intraoperative endoscopic images: from the start of surgery to the first layer of reconstruction. A: The left mucosa of the nasal septum was incised and elevated using a scalpel, B: Nasal septal cartilage was harvested as reconstructive material, C: The sphenoid crest was resected, and the sphenoid sinus was opened wide along the midline, D: Removal of the sphenoid sinus mucosa revealed multiple bony defects. White arrows indicate the bony defects, E: The bone was drilled, and multiple bony defects were joined together to form one large bony defect, F: The space between the bone and dura mater was dissected and clearly identified, G: The bone, dura, and dural defects were made clearly visible, and H: Collagen matrix was implanted in the dural defect in the form of a “bath plug.” CL: clivus; CM: collagen matrix; DU: dura mater; FI: fistula; lSM: left septal mucosa; NS: nasal septum; rSM: right septal mucosa; SC: septal cartilage; SE: sella; SS: sphenoid sinus.

**Figure 4:**
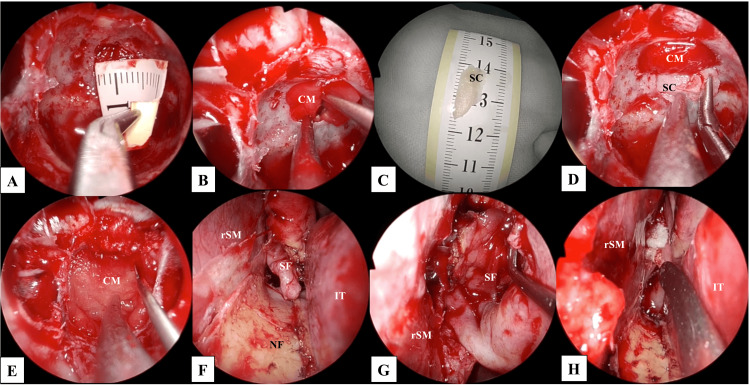
Intraoperative endoscopic images: from the second layer of reconstruction to the end of surgery. A: The size of the bony defect was measured to determine the size of the reconstructive materials required, B: The collagen matrix was present in the layer between the dura mater and bone, C: The border between the nasal septal cartilage and bone was formed as a rigid reconstructive material, D: The formed rigid reconstructive material was inlayed on the layer of the collagen matrix, between the bone and the dura mater, E: The collagen matrix was implanted in the superficial layer of the bony defect, F: A pedicled nasoseptal flap was elevated, extending from the left nasal septum to the floor of the nasal cavity, G: The nasoseptal flap was repositioned to cover the clival defect, H: The nasal septal mucosa on the right side was completely preserved, and the nasal structures were successfully preserved. CL: clivus; CM: collagen matrix; DU: dura mata; IT: inferior turbinate; rSM: right septal mucosa; SC: septal cartilage; SF: septal flap.

Surgery was performed after the improvement of meningitis. Using a transseptal approach, the sphenoid sinus was opened, the sphenoid crest was extensively resected, the septum of the sphenoid sinus was removed (Figures 3A-3C), and the mucosa of the affected left sphenoid sinus was excised. Two bony defect sites that had been detected on the CT images were identified (Figure 3D). The clivus was drilled to merge two small bony defects into one large bony defect (Figures 3E, 3F). The space between the bone and the dura was dissected to create sufficient space for the placement of reconstructive material. Thereby, the dural defect of 2 mm in diameter was identified clearly (Figure 3G). CSF leakage from the dural defect and pulsation of the basilar artery were observed. A collagen matrix (DuraGen^TM^ Dural Graft Matrix (Integra Life Sciences, Plainsboro, NJ, USA)), a type of artificial material, was implanted over the dural defect, with its central part carefully shaped to conform to the defect, serving as a “bath plug” in the first layer (Figure 3H). The size of the bony defect was measured as 8 mm in diameter to determine the size of the rigid reconstruction material required (Figure 4A). The collagen matrix was laid between the dura mater and the bone, as the second layer (Figure 4B). The border between the nasal septal cartilage and bone was designed to be approximately 15 mm by 7 mm and placed between the bone and the second layer to provide a rigid layer (Figures 4C, 4D). Subsequently, a collagen matrix was placed as an overlay against the bone and covered with a pedicled nasal septal flap (Figures 4F-4H). Finally, the surgery was completed with the placement of a lumbar drainage system, necessitated by the relatively high intraoperative CSF outflow.

**Figure 5:**
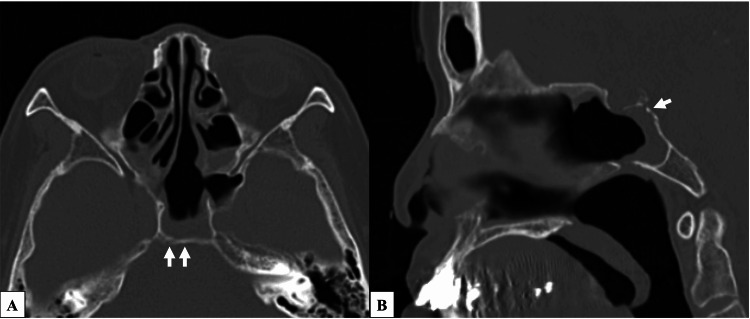
Computed tomography findings around the clivus at one month after surgery. The bony defects were well reconstructed. White arrows indicate the areas where bony defects were identified.

**Figure 6:**
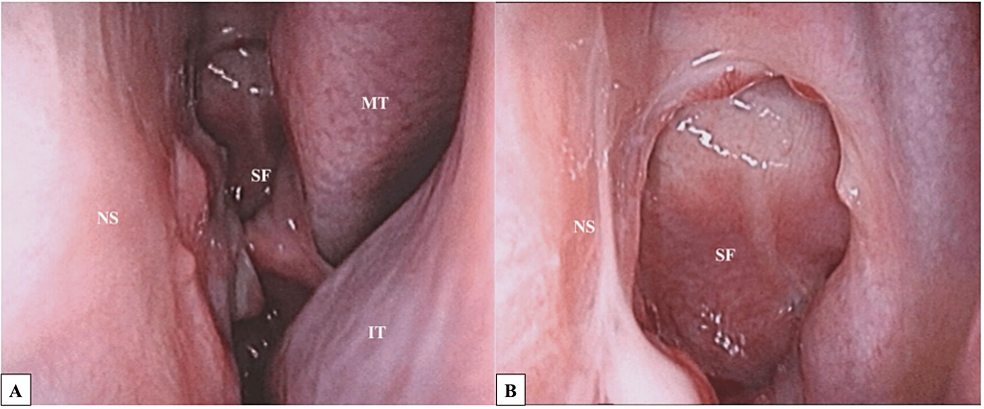
Endoscopic view of the left nasal cavity at two months after surgery. All nasal structures are preserved, and the nasoseptal flap was well engrafted. NS: nasal septum; IT: inferior turbinate; MT: middle turbinate; SF: septal flap.

No findings suggestive of postoperative spinal fluid leakage were encountered; thus, the lumbar drain was removed on the third postoperative day. Owing to the persistent low-grade fever, around 37°C, after surgery, intravenous antimicrobial infusion was continued, but was terminated after fever resolution on the ninth postoperative day. No significant complications were noted, and the patient was discharged on the 14th postoperative day. One month postoperatively, CT images showed that the bony defect in the clivus had been well reconstructed (Figure 5). Two months after surgery, no further nasal crusting was observed. All nasal structures were preserved, and the nasoseptal flap had been well engrafted (Figure 6). At eight months post-surgery, the patient remained in good condition.

## Discussion

In this case, we propose the feasibility of minimally invasive reconstruction surgery for CSF leakage at the clivus, without the need for harvesting autografts at the other operative site, and highlight a novel and effective technique for merging multiple bony defects into a single large defect, facilitating dura defect repair.

For skull base reconstructions due to spontaneous CSF leakage at the clivus, previous studies have reported harvesting autografts and conducting a multilayered reconstruction (Table 1) [1-13]. However, these techniques require invasive procedures outside the main surgical site, i.e., the endonasal area, to harvest the reconstructive material. Conversely, in this case, the use of artificial material allowed for good closure without requiring invasion outside the nasal cavity. Our technique achieved good results with less invasive surgery.

**Table 1:** Previous case reports of spontaneous cerebrospinal fluid leakage from the clivus. EET: endonasal endoscopic transsphenoidal; N/A: not applicable.

Author		Sex	Age	Meningitis before diagnosis	Duration of CSF leak (month)	Defect size (mm)	Approach	Material for reconstruction	Mucosal flap
Vyas et al. [6]	(2022)	M	66	+	48	N/A	EET	None	Nasoseptal flap
Mousavinejad [1]	(2021)	F	36	-	1	N/A	EET	Fascia, fat	Nasoseptal flap
		M	57	+	6	N/A	EET	Fascia, fat	Nasoseptal flap
Ogasaswara et al. [3]	(2021)	F	54	+	15	1.8, 1.0	EET	None	Nasoseptal flap
Nogueira et al. [7]	(2019)	F	48	-	60	N/A	EET	None	Nasoseptal flap
Karli et al. [4]	(2018)	M	56	+	6	7	EET	Fascia, fat, turbinate bone	None
Tandon et al. [8]	(2017)	F	55	+	84	N/A	Sublabial	Fascia, fat	None
Codina Aroca et al. [2]	(2017)	F	52	+	48	2	EET	Fascia	Nasoseptal flap
		M	69	+	N/A	4	EET	Fascia	Nasoseptal flap
Asad et al. [9]	(2017)	F	64	+	288	N/A	EET	Fat	Free mucosal graft
Pagella et al. [10]	(2016)	F	91	-	N/A	N/A	EET	Fascia	Free mucosal graft
		F	36	-	N/A	N/A	EET	Fascia	Free mucosal graft
		F	59	-	N/A	N/A	EET	Fascia	Free mucosal graft
		F	63	+	N/A	N/A	EET	None	Nasoseptal flap
		F	58	+	N/A	N/A	EET	None	Nasoseptal flap
		F	55	-	N/A	N/A	EET	None	Nasoseptal flap
Oleś et al. [11]	(2016)	F	60	+	36	16	EET	Fascia, fat, nasal cartilage	Nasoseptal flap
Hayashi et al. [12]	(2015)	M	38	+	1	N/A	EET	Fascia, fat	Nasoseptal flap
Van Zele et al. [13]	(2013)	F	37	-	2	2.5	EET	Fascia, fat	Free mucosal graft
		F	61	-	4	3.3	EET	Fascia, fat	Free mucosal graft
		F	78	-	3	2	EET	Fascia, fat	Free mucosal graft
		F	42	-	2	1.5	EET	Fascia, fat	Nasoseptal flap
		F	48	-	3	1.8	EET	Fascia, fat	Nasoseptal flap
		M	50	-	1	1.5	EET	Fascia, fat	Nasoseptal flap
Ahmad et al. [5]	(2008)	M	50	+	N/A	N/A	EET	Fascia, fat	None
		M	56	-	24	N/A	Sublabial	Fascia, fat	Free mucosal graft

By merging multiple bony defects into a single large defect, we were able to identify the defect in the dura mater clearly. Additionally, it allowed for the detachment and insertion of reconstructive materials between the bone and dura mater layers. While it led to the drawback of making a large bony defect, it also presented the advantage of allowing for rigid reconstruction. Considering the possible influence of increased intracranial pressure and a pulsating basilar artery on the development of perforation, rigid reconstruction can be considered a suitable method. This method is applicable wherever endonasal endoscopic skull base surgery is possible. Rigid reconstruction has been reported in three patients, including the present case, with bony defects of 7 mm [4], 8 mm, and 16 mm [11] in diameter. Of course, rigid reconstruction should only be attempted in situations where it is feasible. It may be desirable to implant a rigid material larger than the bone defect. This requires ensuring that the layer between the bone and the dura is clearly identified.

Regarding the diagnosis of this condition, half of these patients have a history of meningitis before the diagnosis of CSF leakage [1-6,8-12], and all have serous rhinorrhea [1-9,11-13]. This is a characteristic course of the condition but may be overlooked because of the rarity of the condition itself. In our case, a rhinologist was not present at the time of the initial examination, and hence, the condition was missed. Although the condition is rare, it should be suspected and close examinations, such as thin-slice CT images, MR cisternography, and measurement of glucose levels in nasal secretions, conducted in patients with serous rhinorrhea who have a history of meningitis.

## Conclusions

This case report illustrates that spontaneous CSF leakage from the clivus can be reconstructed in a minimally invasive, without the extranasal autograft harvesting. Additionally, rigid reconstruction can be performed for multiple bony defects, while considering the pathophysiology of the condition. This is considered to be a generalizable surgical procedure because it uses a readily available artificial material. Further similar cases should be accumulated to verify the utility of this approach.
